# YTHDF2-KIF26B-Wnt signaling forms a positive-feedback regulatory loop to maintain intestinal stem cell stemness

**DOI:** 10.1186/s13619-025-00240-2

**Published:** 2025-06-20

**Authors:** Zinan Liu, Chunlin Li, Meimei Huang, Ye-Guang Chen, Yuan Liu

**Affiliations:** 1 The State Key Laboratory of Membrane Biology, Tsinghua-Peking Center for Life Sciences, School of Life Sciences, Tsinghua University, Beijing, 100084 China; 2 The MOE Basic Research and Innovation Center for the Targeted Therapeutics of Solid Tumors, School of Basic Medical Sciences, Jiangxi Medical College, Nanchang University, Nanchang, 330031 China; 3 Guangzhou National Laboratory, Guangzhou, 510700 China

Dear editor,

N6-adenosine methylation (m^6^A) is an important post-transcriptional modification that regulates gene expression through the “writer-reader-eraser” system (Zaccara et al. 2019), which is crucial for the maintenance of intestinal epithelium (Liu et al. 2023). METTL3, as an important “writer” protein, has been reported to play a crucial regulatory role in the stemness and cell death of the small intestinal epithelium (Liu et al. 2023). “Reader” proteins, including YTHDF1/2/3, YTHDC1/2, IGF2BP1/2/3, etc., recognize the m^6^A modification sites and regulate mRNA translation, stability, splicing, and nuclear export (Zaccara et al. 2019). Han and colleagues show that YTHDF1 is crucial for maintaining intestinal stem cells (ISCs) during the processes of regeneration and tumorigenesis (Han et al. 2020), while YTHDC1 deficiency accelerates the development of inflammatory bowel disease (Ge et al. 2023). However, under homeostatic conditions, the functions of YTHDF2 and its specific regulatory mechanisms in small intestinal epithelium are still poorly understood. Here, we found that the “reader” protein YTHDF2 functions as part of a positive-feedback regulatory loop centered around the Wnt signaling pathway. This loop ensures that ISCs remain in a stem-cell state, enabling the intestinal epithelium to function stably and properly.

Based on single cell RNA sequencing (Liu et al. 2023), we identified a distinct expression pattern of *Ythdf2* within the small intestinal epithelium. Notably, *Ythdf2* expression was particularly enriched in the crypt compartment, specifically in ISCs and transit-amplifying (TA) cells (Fig. 1A). Although *Ythdf2* exhibited relatively high-level expression in Tuft cells, ISCs and TA cells showed both the highest average expression and the greatest percentage of expressing cells compared to other epithelial populations. This spatial restriction was further validated at the protein level through immunofluorescence, which confirmed the localization of YTHDF2 in the crypt base (Fig. 1B). These results suggest a potential functional significance of YTHDF2 in maintaining stem cell properties.

**Fig. 1:**
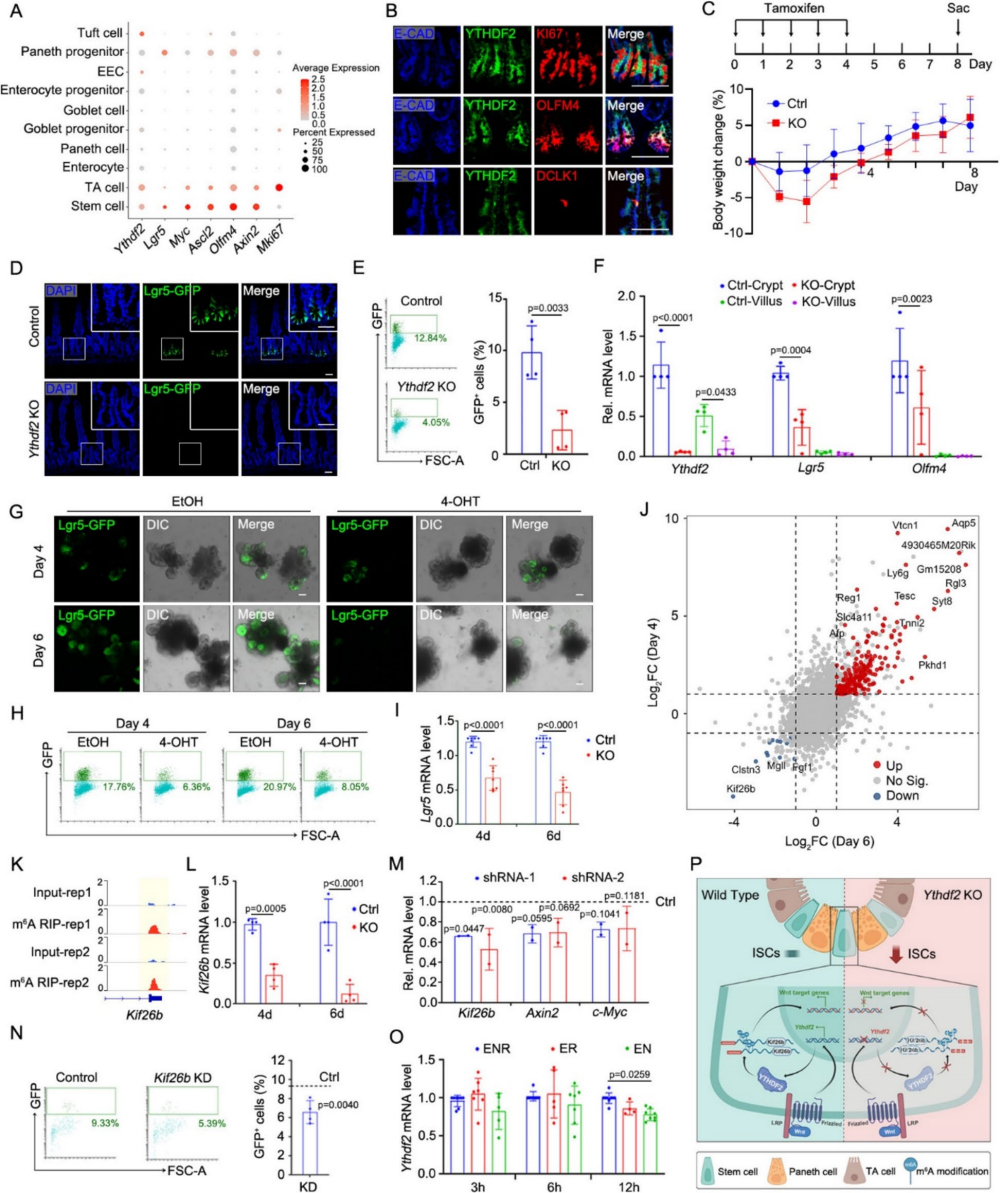
A positive-feedback loop formed by YTHDF2-KIF26B-Wnt signaling maintains the stemness of ISCs. **A** scRNA-seq (GSE186917) revealed *Ythdf2* expression profiles across cell types. **B** Representative images of the expression of *Ythdf2* in TA cells, stem cells and tuft cells. **C** Top: The schematic of mouse experimental design; Bottom: Relative body weight change of *Ythdf2*-KO-GFP mice after treated daily with oil (Ctrl) or tamoxifen (KO) for 5 times. *n* = 5 mice/group. **D** Representative images of Lgr5-GFP^+^ cells in the proximal jejunum of *Ythdf2*-KO-GFP mice after treated daily with oil (Control) or tamoxifen (*Ythdf2* KO) for 5 times at day 8. Nuclei were counter-stained with DAPI. *n *= 3 mice/group. **E** FACS analysis result and quantification of Lgr5-GFP^+^ cells in crypts at day 8. *n *= 4 mice/group. **F** qRT-PCR result of *Ythdf2* and ISCs marker genes. *n *= 4 mice/group. **G-I** Representative images (**G**), FACS analysis (**H**) and qRT-PCR (**I**) result of *Lgr5* expression in *Ythdf2*-KO-GFP organoids at day 4 or 6 after treatment with EtOH (Ctrl) or 4-OHT (KO). *n *> 3 repeats/group. **J** Scatter plot of isolated Lgr5^+^ ISCs from control, *Ythdf2*-KO-4d, and *Ythdf2*-KO-6d cultured organoids. |log_2_FC|≥ 1; adjusted *p*-value < 0.05. **K** IGV tracks displaying MeRIP-seq (GSE186917) reads along *Kif26b* in Lgr5^high^ ISCs. The Y axis represents the CPM (count per million) of genes. The yellow boxes of the tracks depict the positions of m^6^A peaks. **L** qRT-PCR result of *Kif26b* expression in *Ythdf2*-KO-GFP organoids. *n *= 4 repeats/group. **M** Expression of *Kif26b* and Wnt signaling marker genes in control and *Kif26b*-knockdown organoid. shRNA-1/2 represents infection of lentiviruses carrying two different shRNAs both targeting *Kif26b*, respectively. Dashed line: expression profile in wild-type organoids infected with control shRNA. *n *= 2 repeats/group. **N** Representative FACS analysis result and quantification of Lgr5-GFP^+^ cells in control and *Kif26b*-KD organoid. Dashed line: percentage of Lgr5-GFP^+^ cells in wild-type organoids infected with control shRNA. *n *= 4 different shRNAs. **O** *Ythdf2* expression in organoids at indicated time points after Noggin or R-spondin withdrawal. *n *> 3 repeats/group. **P** A Schematic overview. All the data represent mean ± SD. The data were analyzed by Two-way ANOVA (**F, I, L, M, O**) and unpaired two-tailed t-test (**E, N**). The exact *P *value is displayed. Scale bars: 50 μm (**B**, **D**, **G**)

To elucidate the function of YTHDF2 in the small intestinal epithelium and observe its effect on Lgr5^+^ ISCs, we generated *Villin-CreERT2; Lgr5-EGFP-IRES-CreERT2; Ythdf2*^*fl/fl*^ (*Ythdf2*-KO-GFP) mice. Although the body weight and the length of the mouse intestine in *Ythdf2*-KO-GFP mice did not change after tamoxifen injection (Fig. 1C and S1A), tamoxifen-induced deletion of *Ythdf2* significantly decreased Lgr5^+^ ISCs through confocal microscopy and fluorescence-activated cell sorting (FACS) analysis (Fig. 1D-E). In addition, the qRT-PCR experiment also revealed that the knockout of *Ythdf2* could induce a down-regulation in the expression of stemness genes *Lgr5* and *Olfm4* (Fig. 1F). Meanwhile, cell differentiation was not affected by *Ythdf2* KO as goblet cell, Paneth cells, and enteroendocrine cells did not change compared with the control group through qRT-PCR analysis and immunofluorescence staining (Fig. S1B-C). Consistently, the impairment of ISCs was verified in 4-hydroxytamoxifen (4-OHT)-induced *Ythdf2* KO organoids from day 4 to day 6 (Fig. 1G-H). In line with this, the expression of *Lgr5* detected by qRT-PCR also revealed that *Ythdf2* deficiency resulted in reduction of Lgr5^+^ cells (Fig. 1I). While, there was a certain degree of recovery in the loss of ISCs one month post tamoxifen injection (Fig. S1D-F). These experimental results indicate that *Ythdf2* deletion leads to small intestinal stem cells loss.

To uncover how *Ythdf2* acts in ISCs, we performed bulk RNA-sequencing in isolated Lgr5^+^ ISCs of control, *Ythdf2*-KO-4d, and *Ythdf2*-KO-6d cultured organoids (Fig. 1J, S2A). The result shows that *Ythdf2* knockout not only downregulates ISC signature genes (Muñoz et al. 2012) but also downregulates proliferation-related genes as well as upregulates genes related to differentiation, apoptosis and regeneration in Lgr5^+^ ISCs at day 4 and day 6 post 4-OHT treatment (Fig. S2B-D). Notably, apoptotic pathways were significantly more dysregulated compared to proliferation, differentiation, or regeneration programs, suggesting ISCs may eventually undergo apoptosis by day 6 post-tamoxifen treatment (Fig. S2D). Interestingly, from the sequencing results, we noticed the downregulation of the gene *Kif26b* (Fig. 1J) and found that it has a similar expression pattern to *Ythdf2* in the small intestinal epithelium (Fig. 1A-B and Fig. S2E). To explore the relationship among YTHDF2, KIF26B and m^6^A modification, we have re-analyzed our previous m^6^A-seq data and transcriptional data from *Mettl3*-deleted ISCs (Liu et al. 2023), which could represent m^6^A modification directly. *Kif26b* exhibited low mRNA expression in *Mettl3*-KO ISCs (Fig. S2F), highly implicating m^6^A modification in regulating *Kif26b* expression. In addition, *Ythdf2* KO led to a significant decrease in *Kif26b* expression (Fig. S2G), particularly at *Kif26b* gene loci harboring abundant m^6^A sites (Fig. 1K). Collectively, the above results support that *Kif26b* mRNA stability relies on m^6^A modification, which is catalyzed by METTL3 and recognized by YTHDF2, to maintain its stability. The decreased expression of *Kif26b* was further validated by qRT-PCR, with the magnitude of downregulation becoming more pronounced from day 4 to day 6 (Fig. 1L). It has been reported that YTHDF2 could bind to the mRNA of *Kif26b* (Chen et al. 2022; Wang et al. 2013) and knockdown of *Kif26b* can inhibit the activation of the Wnt signaling pathway (Yan et al. 2020). Here, we found that *Kif26b* knockdown or knockout could downregulate *Axin2* and *c-Myc* in organoids (Fig. 1M, Fig. S2H), which are the target genes of the Wnt signaling pathway, and the results of FACS analysis also showed that *Kif26b* reduction led to a decrease in the number of Lgr5^+^ ISCs (Fig. 1N). Taken together, these data indicate that *Ythdf2* regulates the Wnt signaling pathway via KIF26B, thereby maintaining Lgr5^+^ stem cells.

Given the characteristic that *Ythdf2* has a high expression pattern in stem cells and TA cells, we hypothesized that *Ythdf2* could sustain its own expression pattern through a regulatory feedback loop. Therefore, we systematically modulated key signaling pathways in cultured organoids by removing Noggin and R-spondin, which inhibit BMP signaling pathway and enhance the Wnt signaling pathway, respectively. Notably, the removal of R-spondin led to a more significant decrease in *Ythdf2* expression, indicating the Wnt signaling regulates the expression of *Ythdf2* in the small intestinal epithelium (Fig. 1O).

In summary, we found that YTHDF2, an important m^6^A “reader” protein, regulates the expression of stemness genes by modulating the Wnt signaling in ISCs through KIF26B. Meanwhile, YTHDF2 is regulated by the Wnt pathway, forming a positive-feedback loop to maintain the homeostasis of the small intestinal epithelium (Fig. 1P). Building on prior work elucidating the role of METTL3/YTHDF1/YTHDC1 in intestinal biology, our study advances the intricate mechanisms and provides granular insights into the “reader”-specific mechanisms of m^6^A-mediated ISC regulation, thereby completing the functional triad of m^6^A regulatory machinery. While, we also found that the impact of *Ythdf2* knockout on ISCs can be restored after a long time. It suggests compensatory mechanism may be engaged, potentially through YTHDF1/YTHDF3 upregulation, which is consistent with previously functional redundancy of YTHDF proteins (Zaccara and Jaffrey 2020). Furthermore, recent research proposes that YTHDF2 stabilizes target mRNAs by recognizing m^5^C in the tumor immunity (Chen et al. 2025), highlighting an alternative compensatory regulatory mechanism expect for m^6^A modification. In addition, despite evidence that YTHDF2 induces target mRNA degradation in human systems (Wang et al. 2013), our *Ythdf2* deletion experiments revealed reduced KIF26B expression. This paradoxical finding suggests that YTHDF2 may maintain the stability of *Kif26b* through some mechanism that is not yet fully understood. This regulatory mechanism may also exhibit species and tissue specificity, implying significant differences in post-transcriptional regulation among different species, which could be a direction warranting further investigation.

## Supplementary Information

Supplementary Material 1. Supplementary Methods. Fig. S1: *Ythdf2* knockout exhibits no significant effects on length of intestine or differentiated lineage. Fig. S2: *Kif26b* and ISC significant genes were downregulated upon Ythdf2 deletion. Table S1: The list of shRNA or sgRNA sequence. Table S2: qRT-PCR primer used in this study.

Supplementary Material 2. Table S3: Gene lists of signature profile and its expression level at the indicated time upon *Ythdf2* deletion, related to Fig. S2D.

## Data Availability

The RNA-seq data generated in this study are publicly available through the Gene Expression Omnibus with the accession code GSE290302. Data from GSE186917 was used to analyze the expression pattern of *Ythdf2* through scRNA-seq data and the m^6^A modification motif of *Kif26b* through MeRIP-seq data. All codes that the main steps of the analysis and data are available from the corresponding author under request.

## Abbreviations

4-OHT: 4-Hydroxytamoxifen
AAV: Adeno-associated virus
Alpi: Alkaline phosphatase, intestinal
Ascl2: Achaete-scute family bHLH transcription factor 2
Chga: Chromogranin A
c-Myc: Cellular myelocytomatosis oncogene
Ctrl: Control
DAPI: 4',6-Diamidino-2-Phenylindole
Dclk1: Doublecortin-like kinase 1
Down: Downregulated
EEC: Enteroendocrine cell
EtOH: Absolute ethanol
IGF2BP1/2/3: Insulin-like growth factor 2 mRNA-binding protein 1/2/3
IGV: Integrative Genomics Viewer
ISC: Intestinal stem cell
KD: Knockdown
KIF26B: Kinesin-like protein KIF26B
KO: Knockout
Lgr5: Leucine rich repeat containing G protein coupled receptor 5
LI: Large intestine
Lyz: Lysozyme
m^6^A: N6-adenomethylation
MeRIP-seq: Methylated RNA immunoprecipitation sequencing
METTL3: Methyltransferase-like 3
Muc2: Mucin 2
NO Sig.: Non-significant
Olfm4: Olfactomedin 4
Sac: Sacrificed
scRNA-seq: Single-cell RNA sequencing
SI: Small intestine
TA: Transient amplifying
TAM: Tamoxifen
TSA: Tyramide signal amplification
Up: Upregulated
YTHDC1/2: YTH domain-containing protein 1/2
YTHDF1/2/3: YTH domain-containing family protein 1/2/3
