# Supplementary material for: YTHDF2-KIF26B-Wnt signaling forms a positive-feedback regulatory loop to maintain intestinal stem cell stemness
Source: Cell Regen. 2025 Jun 20;14:26. doi: 10.1186/s13619-025-00240-2 (PMC12181566; doi:10.1186/s13619-025-00240-2)
Supplement: Supplementary file 1 — Supplementary Material 1. Supplementary Methods. Fig. S1: Ythdf2 knockout exhibits no significant effects on length of intestine or differentiated lineage. Fig. S2: Kif26b and ISC significant genes were downregulated upon Ythdf2 deletion. Table S1: The list of shRNA or sgRNA sequence. Table S2: qRT-PCR primer used in this study. [file 13619_2025_240_MOESM1_ESM.docx]

**YTHDF2-KIF26B-Wnt signaling forms a positive-feedback regulatory loop to maintain ISC stemness**

Zinan Liu^1^, Chunlin Li^1^, Meimei Huang^1,3^, Ye-Guang Chen^1,2,3,#^, Yuan Liu^1,#^

Supplementary Method

Supplementary Figures: S1-S2

Supplementary Table: S1-S3

**Supplementary Methods**

**Mice.**

*Ythdf2^fl/fl^* mice were from Dr. Meng Xu (Tsinghua University), *Lgr5-EGFP-IRES-CreERT2* mice were obtained from the Jackson Laboratory, and *Vil-CreERT2* mice were a gift from Dr. Sylvie Robine (Institut Curie-CNRS). Above mice were crossed to generate *Villin-CreERT2; Lgr5-EGFP-IRES-CreERT2; Ythdf2^fl/fl^* (*Ythdf2*-KO-GFP) mice. The *Ythdf2*-KO-GFP mice were intraperitoneally injected with 5 consecutive daily doses of 20 mg/mL tamoxifen (TAM) (Sigma, T5648-5G) in sunflower oil to induce *Ythdf2* knockout. In the control group, mice of the same genotype were injected with oil at the same mass-volume fraction. All animal studies were conducted in accordance with the relevant guidelines and under the approval of the Institutional Animal Care and Use Committee of Tsinghua University.

**Isolation of intestinal crypts and organoid culture.**

Mouse small intestinal crypts were isolated and cultured according to the established protocol as previously described (Liu et al., 2023). The enriched intestinal crypts were embedded in Matrigel (Corning, #356231) and seeded onto a 24-well plate and cultured in ENR medium. This medium consisted of Advanced DMEM/F12, along with EGF (50 ng/mL, Invitrogen), Noggin (100 ng/mL, OrganRegen), R-spondin1 (500 ng/mL, OrganRegen), Penicillin/Streptomycin, GlutaMAX-I, N2, B27, and N-acetylcysteine (Sigma-Aldrich). To induce *Ythdf2* knockout, 0.5 μM 4-Hydroxytamoxifen (4-OHT) was added to the ENR culture medium for 2 days while an equal volume of absolute ethanol (EtOH) was added to the medium in the control group.

**Virus production and organoid infection.**

Methods for the production of lentivirus and recombinant adeno-associated virus (AAV), as well as their infection of organoids, were carried out as previously described (Liu et al., 2023). *Villin-CreERT2; Lgr5-EGFP-IRES-CreERT2; Ythdf2^fl/fl^* organoids were prepared for lentivirus and AAV infection*,* which contain shRNA of *Kif26b*. Prior to virus infection, the organoids were cultured in an expansion medium (ENR medium containing 6.67 μM blebbistatin, 2.5 μM CHIR-99021 and 10% Wnt3a conditional medium) supplemented with 10 mM nicotinamide for two days. After that, TrypLE (Gibco, 12604021) was used to digest the organoids into a cell pellet. The cell pellet was then resuspended in the expansion medium containing 10 μg/ml polybrene (Macgene, MC032) and mixed with the virus. Subsequently, 250 μL of the cell-virus mixture in the expansion medium with polybrene was added to the pre-solidified Matrigel and cultured overnight. On the next day, the medium was removed, and the virus was washed away with warm PBS. After the washing step, a 10 μL overlay of Matrigel was applied, and the organoids were cultured in the expansion medium. The culture medium was replaced with ENR supplemented with 2 μg/mL puromycin at day 2 post-infection. Two types of lentiviruses respectively contain shRNA-1 and shRNA-2, while six kinds of AAVs respectively contain shRNA-1, 2, 3, 4 and sgRNA-1, 2. shRNA and sgRNA sequences are listed in Supplementary Table S1.

**Immunofluorescence.**

Immunofluorescence and (TSA)-mediated immunofluorescence were performed as previously described (Liu et al., 2023). The small intestine was isolated from mice and washed several times with cold PBS. Subsequently, it was fixed with a 4% formaldehyde solution. After dehydration in 20% sucrose overnight, the tissue was embedded in OCT (Sakura). Sections were prepared using a freezing microtome (Leica) and then observed under an Olympus FV3000 confocal microscope. All sections and organoids were carried out with a 20×magnification setting on the Olympus 20×/0.3 air objective. Sections were incubated with DAPI at room temperature for 1h. For YTHDF2 staining, mouse anti-YTHDF2 antibody (1:300, Abcam, ab246514) was used and the signals were amplified by TYR-520 dye of TSA kit (Recordbio Biological Technology, Cat#RC0086-1) following the manufacturer’s instructions. Other primary antibodies for immunofluorescence were mouse anti-E-cadherin (1:500, BD Transduction Laboratories, 610182), rabbit anti-Ki67 (1:300, Abcam, ab15580), rabbit anti-Olfm4 (1:500, CST, 19141), rabbit anti-DCAMKL1 (1:300, Abcam, ab109029), rabbit anti-Lysozyme (1:300, Abcam, ab108508), rabbit anti-Chromogranin A (1:300, Abcam, ab15160), rabbit anti-MUC2 (1:300, Abcam, ab272692). Secondary antibodies were Alexa Fluor® 488 AffiniPure™ Donkey Anti-Mouse IgG (H+L) (1:1000, Jackson, 715-545-150), Alexa Fluor® 647 AffiniPure™ Donkey Anti-Mouse IgG (H+L) (1:1000, Jackson, 715-605-150) and Rhodamine (TRITC) AffiniPure™ Donkey Anti-Rabbit IgG (H+L) (1:1000, Jackson, 711-025-152).

**Flow cytometry.**

To obtain a single-cell suspension, crypts from *Ythdf2*-KO-GFP on day 8 after oil or TAM injection as well as control, *Ythdf2*-KO-4d, and *Ythdf2*-KO-6d cultured organoids were incubated in TrypLE (Gibco, 12604021) at 37 ℃ for 20 minutes. The dissociated cells were filtered through a 40μm cell strainer (BD) and then stained with 4',6-Diamidino-2-Phenylindole (DAPI, Invitrogen, D1306). ISCs (DAPI^-^GFP^+^) were analyzed (CytoFlex LX, Beckman) or sorted by flow cytometry (BD FACS AriaII).

**Quantitative real-time PCR (qRT-PCR)**.

The RaPure Total RNA Kit (Magen, R4011) was utilized to extract total RNA from tissues or organoids. Subsequently, cDNA was synthesized by means of the cDNA Synthesis SuperMix (Novoprotein, E047-01A). qRT-PCR was performed in triplicate on a LightCycler 480 (Roche). The reaction employed NovoStart®SYBR qPCR SuperMix Plus (Novoprotein, E096-01A), with *Gapdh* serving as the reference gene. Data analysis was carried out in accordance with the 2^-ΔΔCT^ method. The sequences of the primers used are listed in Supplementary Table S2.

**Bulk RNA-seq and analysis.**

Total RNA was extracted with RNeasy Mini Kit (QIAGEN, 74104) according to the manufacturer’s instruction. Bulk RNA-seq was performed using the Illumina Hiseq X Ten platform and analyzed as previously described (Liu et al., 2023). Bulk RNA sequencing reads were aligned to the mouse genome reference (GRCm39) using STAR (Version 2.7.10b) with default parameters. Genes were considered significantly differentially expressed if they had a Benjamini-Hochberg (BH) adjusted p-value < 0.05 and a log2 fold change (logFC) greater than 1 (upregulated) or less than -1 (downregulated). Differentially expressed genes were identified using EdgeR (Version 4.0.16) and DESeq2 (Version 1.42.1) software. GSEA was conducted using the clusterProfiler (Version 4.10.1) with default settings. ISC signature genes as well as proliferation, differentiation, apoptosis and regeneration-related genes were utilized for GSEA analysis (Liu et al., 2023; Muñoz et al., 2012).

**Methylated RNA immunoprecipitation (MeRIP)-seq and Single cell RNA sequencing (scRNA-seq) analysis.**

A single-cell RNA sequencing (scRNA-seq) dataset (GSE186917) (Liu et al., 2023) of the intestinal epithelium was used. We detected the expression of *Ythdf2* and *Kif26b* in the original processing results of the wild-type scRNA-seq library and visualized the expression of *Ythdf2*. Additionally, we conducted visualizations of m^6^A level of *Kif26b* using the original processing results obtained from MeRIP-seq.

**Statistics.**

All experiments were independently repeated. Except for Fig. 1M, which was repeated only twice, at least three biological replicates were set. Statistical analysis was performed using Graphpad Prism (Version 8), and schematic overview was created with BioRender.com. The data presented in the figures represent the mean±standard deviation, as specified in the figure legends. As indicated in the figure legends, unpaired two-tailed t-test and two-way ANOVA were used to compare differences.

**
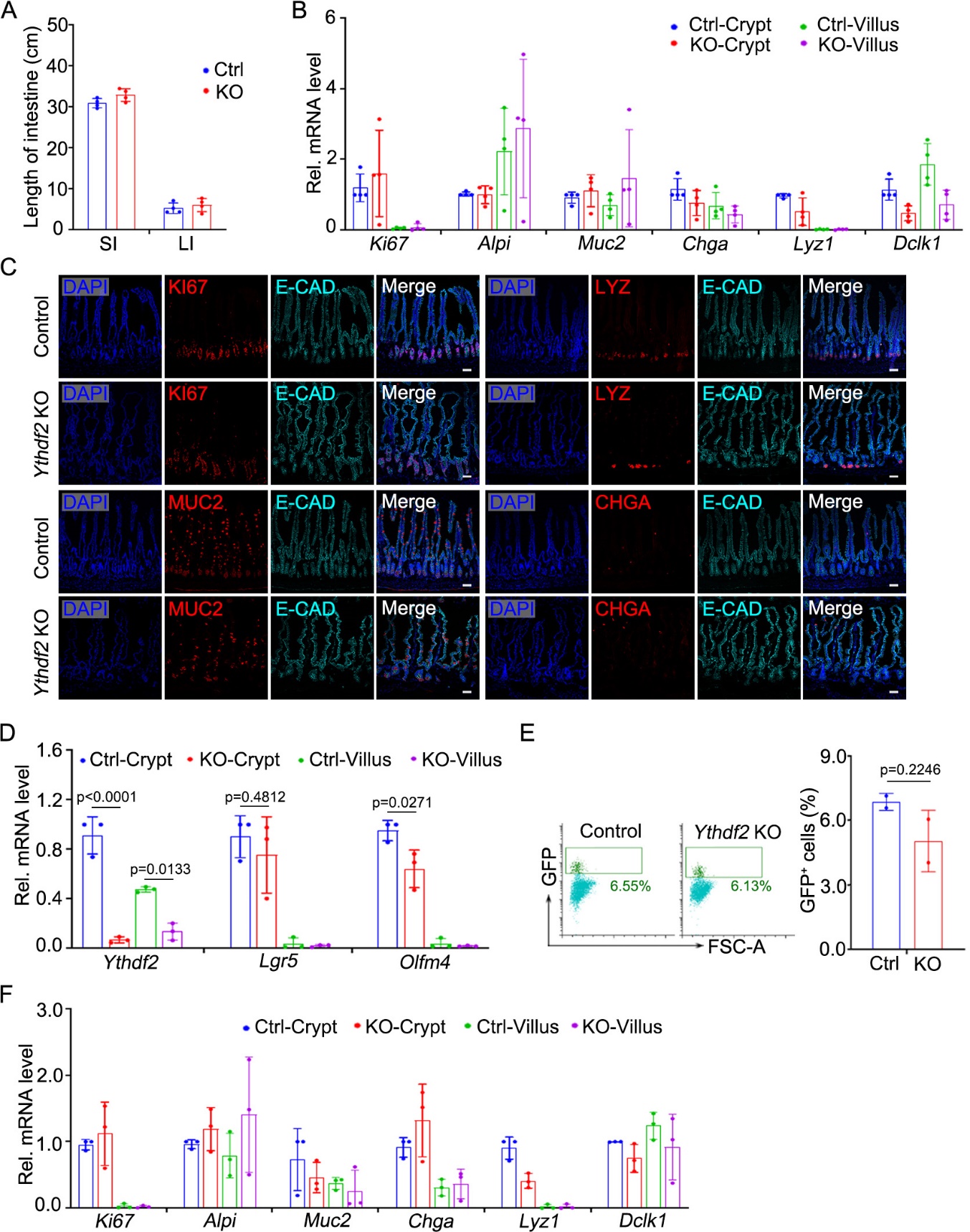
Fig. S1** *Ythdf2* knockout exhibits no significant effects on length of intestine or differentiated lineage. **A** The length of small intestine (SI) and large intestine (LI) in control and KO mice at day 8 after tamoxifen injection. n= 4 mice/group. **B** Expression level of differentiated cells (TA cell-*Ki67*, enterocyte-*Alpi*, goblet cell-*Muc2*, EEC-*Chga*, Paneth cell-*Lyz1*, Tuft cell-*Dclk1*) in crypt or villus from control and KO mice at day 8 after tamoxifen injection. n= 4 mice/group. **C** Representative images of differentiated cells marker staining in small intestinal sections from control and KO mice, collected at day 8 after oil or tamoxifen injection. **D** Expression level of *Ythdf2* and ISC marker genes in crypt or villus from control and KO mice one month post-tamoxifen injection. n=3 mice/group. **E** FACS analysis and quantification of Lgr5-GFP^+^ cells in crypts from control and KO mice one month post-tamoxifen injection. n=2 mice/group. **F** Expression level of differentiated cells in crypt or villus from control and KO mice one month post-tamoxifen injection. n= 3 mice/group. All the data represent mean±SD. All the data are presented as mean±SD. The data were analyzed by Two-way ANOVA (**A**, **B, D, F**) and unpaired two-tailed t-test (**E**). Scale bars: 50 μm (**C**).

**
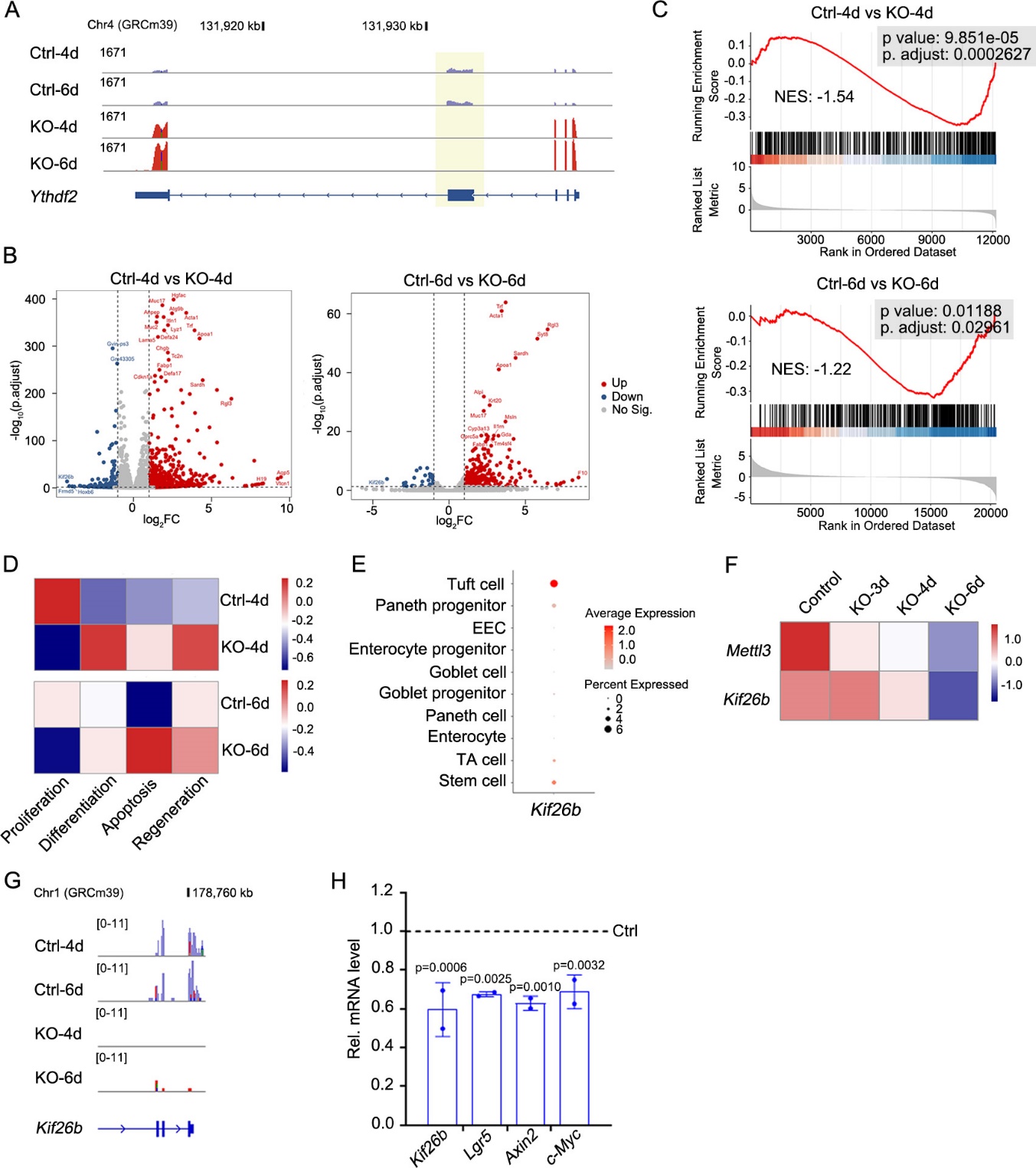
Fig. S2** *Kif26b* and ISC significant genes were downregulated upon *Ythdf2* deletion. **A** Integrative genomics viewer (IGV) of *Ythdf2* gene knockout strategy in the Ctrl-4d, Ctrl-6d, KO-4d and KO-6d groups. **B** Volcano plots depicting differentially expressed genes (DEGs) between Ctrl-4d vs KO-4d, and Ctrl-6d vs KO-6d. Genes that are significantly upregulated are shown in red, downregulated genes in blue, and non-significant genes in gray. The significance threshold is set at an adjusted p-value<0.05 and |log2FC|>1. **C** GSEA analysis of ISC signature gene sets in *Ythdf2*-KO versus control cells. p-value<0.05; 1,000 permutations. Genes were ranked in descending order based on their log_2_FC values, which were calculated from the differential expression between the groups. **D** Heatmap result of proliferation, differentiation, apoptosis and regeneration related gene sets in ISCs in Ctrl-4d, Ctrl-6d, KO-4d and KO-6d groups. **E** scRNA-seq (GSE186917) revealed *Kif26b* expression profiles across cell types. **F** Heatmap result of the expression levels of *Mettl3* and *Kif26b* in ISCs three, four, and six days after the induced knockout of *Mettl3*. **G** IGV demonstrates the changes in the expression level of *Kif26b* in ISCs four days and six days after the induced knockout of *Ythdf2*. **H** Expression of *Kif26b* and Wnt signaling marker genes in control and *Kif26b*-knockout organoid. sgRNA represents infection of AAV carrying two different sgRNAs both targeting *Kif26b*, respectively. Dashed line: expression profile in control organoids. n=2 different sgRNAs. All the data are presented as mean±SD. The data were analyzed by Two-way ANOVA (**H**).

**Table S1** The list of shRNA or sgRNA sequence

| Species | Gene | ID | OLIG_SEQ |
| --- | --- | --- | --- |
| Mouse | *Kif26b* | shRNA-1 | CCGGCCTGCTCATCTTATCTGAGATCTCGAGATCTCAGATAAGATGAGCAGGTTTTTG |
|  |  | shRNA-2 | CCGGCCACTACGAATGCTTGTCGTTCTCGAGAACGACAAGCATTCGTAGTGGTTTTTG |
|  |  | shRNA-3 | CCGGCCAAACATTGTGTCCGTACTTCTCGAGAAGTACGGACACAATGTTTGGTTTTTG |
|  |  | shRNA-4 | CCGGCAGAGTACAAACCTCCCAGTTCTCGAGAACTGGGAGGTTTGTACTCTGTTTTTG |
|  |  | sgRNA-1 | ATGATCCCCTGACTTGCGGAGG |
|  |  | sgRNA-2 | GAGGTATCTCGAGCTGATGTGG |

**Table S2** qRT-PCR primer used in this study

| Gene | Primer sequence |
| --- | --- |
| ***Gapdh-*F** | AAGAAGGTGGTGAAGCAG |
| ***Gapdh-*R** | TCATACCAGGAAATGAGC |
| ***Ythdf2*-F** | GCCATGTCAGACTCCTACTTAC |
| ***Ythdf2*-R** | CCGTTGCTCAGTTGTCCATA |
| ***Kif26b*-F** | CCTGCTTTGACATCACCTCCA |
| ***Kif26b*-R** | GAAACATCGCCTTTCGCTCC |
| ***Lgr5*-F** | CGGGACCTTGAAGATTTCCT |
| ***Lgr5*-R** | GATTCGGATCAGCCAGCTAC |
| ***Ki67*-F** | AGGCTCCGTACTTTCCAATTC |
| ***Ki67*-R** | CGTCTTAAGGTAGGACTTGCAG |
| ***Olfm4*-F** | CGAGACTATCGGATTCGCTATG |
| ***Olfm4*-R** | TTGTAGGCAGCCAGAGGGAG |
| ***Axin2*-F** | GCTCCAGAAGATCACAAAGAGC |
| ***Axin2*-R** | AGCTTTGAGCCTTCAGCATC |
| ***cMyc*-F** | GCTGTTTGAAGGCTGGATTTC |
| ***cMyc*-R** | GATGAAATAGGGCTGTACGGAG |
| ***Muc2*-F** | TGTGGTCTGTGTGGGAACTTTG |
| ***Muc2*-R** | GCTTACATCTGGGCAAGTGGAA |
| ***Chga*-F** | GCTGGAACATAAGCAGGAGG |
| ***Chga*-R** | ATCCTGCTCCATCGCTTG |
| ***Alpi*-F** | GTCCCACCGCTGGTTACTTT |
| ***Alpi*-R** | CTGTGGGCTGAGATGATGTC |
| ***Lyz1*-F** | ACGAGCTACAAACTACAACCG |
| ***Lyz1*-R** | GATCTCTCACCACCCTCTTTG |
| ***Dclk1*-F** | CAGCAAGTCTCCCAGAAGATAC |
| ***Dclk1*-R** | GGACTGTGTAACAGGAGTGAAA |

**Table S3** Gene lists of signature profile and its expression level at the indicated time upon *Ythdf2* deletion, related to Fig. S2D. (Excel file)
